# The Efficacy and Safety of Anlotinib Plus Etoposide with Cisplatin/Carboplatin in the First-Line Treatment of Lung Cancer: A Phase II Clinical Study

**DOI:** 10.7150/jca.91701

**Published:** 2024-05-05

**Authors:** Xiao-ming Lv, Yang Liu, Yan Feng, Hong-liang Liang, Wei-wei Zhi

**Affiliations:** 1 Department of Thoracic Surgery, Linfen Central Hospital, Linfen, Shanxi, 041000, China.; 2 Department of Cardiovascular Surgery, Xijing Hospital, Air Force Military Medical, University, Xincheng, Xi'an, 710032, China.; 3 Department of medical, Xi'an Fourth Hospital, Xincheng, Xi'an, 710004, China.; 4 Department of Cardiovascular Surgery, Xi'an Fourth Hospital, Xincheng, Xi'an, 710004, China.

**Keywords:** Anlotinib, Etoposide, Clinical trials, Lung cancer

## Abstract

**Background:** The primary aim of this phase II clinical study was to assess the safety and efficacy of combining anlotinib, etoposide, and platinum-based drugs as a first-line treatment for ES-SCLC.

**Methods:** Patients underwent the standard chemotherapeutic regimen, consisting of four courses of etoposide plus cisplatin/carboplatin. Additionally, each patient received a 2-week intervention with anlotinib (12 mg/day, once daily). Anlotinib was continued until disease progression, occurrence of unbearable adverse events (AEs), or withdrawal from the research. Progression-free survival (PFS) served as the primary prognostic measure. Secondary measures included the disease control rate (DCR), objective response rate (ORR), overall survival time (OS), and the incidence of AEs.

**Results:** The DCR and ORR were 97.6% and 91.0%, respectively. Estimated PFS and OS were 5.0 months (95% CI: 1.0-10.8 months) and 13.0 months (95% CI: 8.4-18.6 months), respectively. No unexpected adverse effects were reported during the trial. The most common adverse reactions included anemia (42.22%), hypertension (53.33%), alopecia (40.00%), elevated transaminase (24.40%), and elevated alkaline phosphatase (24.44%). Sixteen cases (35.56%) were classified as AEs of grades 3-5. No deaths attributed to treatment-related causes occurred in any patient during the trial.

**Conclusion:** Combination chemotherapy is currently the first-line therapy for extensive small-cell lung cancer (ES-SCLC). Combining anlotinib with conventional platinum-based chemotherapy demonstrated promising therapeutic outcomes and prognosis in the management of ES-SCLC.

## Introduction

A particular form of lung cancer, clinically referred to as small cell lung cancer (SCLC), belongs to the category of highly malignant neuroendocrine tumors, constituting approximately 15% of all lung cancers. The tumor cells exhibit rapid multiplication and a propensity for invasive growth and distant metastasis 1, 2. SCLC primarily manifests in individuals who smoke. It is a malignancy that tends to metastasize widely in its early stages, resulting in relatively short patient survival times. Although responsive to chemotherapy and radiotherapy, it is prone to secondary resistant relapses despite high remission rates during initial treatment. Currently, systemic chemotherapy forms the foundation of treatment 3, 4. Due to its robust up-regulation and early metastasis, approximately 70% of patients receive an initial diagnosis of extensive-stage small cell lung cancer (ES-SCLC), with a median overall survival (OS) of only ten months 5.

When patients are diagnosed with SCLC, approximately 30% are present with localized tumors, while the majority are already in the extensive stage, characterized by the tumor spreading beyond the supraclavicular region 6. In comparison to several other types of lung cancer, chemotherapy and radiotherapy are less effective for treating SCLC 7-10. However, due to the widespread distribution of SCLC at the time of diagnosis, achieving a cure is often challenging 11, 12. Various techniques utilizing artificial intelligence or CT scans may efficiently and promptly detect this form of lung cancer 13-16.

Recently, the standard primary therapy for patients with SCLC has been the combination chemotherapy of etoposide and platinum 3, 17. Despite the initially high response rate, a significant number of patients experience rapid progression. More recently, the addition of immune checkpoint inhibitors (such as atezolizumab or bevacizumab) to platinum-etoposide therapy in ES-SCLC patients has shown a transformative effect on OS, leading to a significant paradigm shift in the primary treatment model for SCLC 3, 17.

Angiogenesis plays a crucial role in tumor development. A promising therapeutic strategy for SCLC involves targeting endothelial cells therapeutically. Bevacizumab, which blocks VEGF, has shown an extended progression-free survival (PFS) when combined with platinum-etoposide chemotherapy for the treatment of ES-SCLC. However, this improvement is observed primarily in PFS rather than OS 18, 19. In comparison to other angiogenesis class inhibitors, Patina, Zivlivet, Pazopanib, and sunitinib have demonstrated potential for maintenance therapy or as second-line medical interventions in SCLC. However, there are no reports indicating their efficacy in improving the survival of previously untreated patients 20, 21.

Anlotinib, a small molecule tyrosine kinase inhibitor currently administered orally to patients, has demonstrated potent antiangiogenic and antitumor effects by targeted inactivation of vascular endothelial growth factor receptor (VEGFR), fibroblast growth factor receptor (FGFR), and platelet-derived growth factor receptor (PDGFR) 4, 22. Anlotinib, independently registered for both first- and third-line relapsed SCLC in China, has demonstrated significant improvements in OS and PFS. Another study has also shown similar clinical benefits of anlotinib in recurrent cases of SCLC, highlighting its remarkable efficacy and safety in the treatment of this cancer subtype 23-25.

The integration of anlotinib, a potent small-smolecule tyrosine kinase inhibitor targeting VEGFR, FGFR, and PDGFR, into first-line treatment regimens alongside etoposide and platinum-based chemotherapy presents a novel approach with promising therapeutic outcomes in ES-SCLC 26, 27. Therefore, this trial aims to evaluate the efficacy of combining etoposide and platinum with anlotinib in ES-SCLC and assess any associated adverse effects.

## Methods

### Main indicators of clinical trial evaluation and ethical review

The research project was conducted at Linfen Central Hospital from February 2019 to February 2021. The evaluated performance indicators included the objective control rate (ORR), OS, adverse events (AEs), and disease control rate (DCR). PFS was the primary metric for assessing outcomes. Ethical approval for this study was granted by the Linfen Central Hospital's ethics committee. Prior to participation, written informed consent was obtained from each participant. All procedures were carried out in strict adherence to applicable guidelines and regulations.

### Exclusion and inclusion criteria of clinical research subjects

Inclusion criteria: (1) Adult patients (over 18 years old but under 75 years old); (2 Histological or cytological clinical diagnosis of ES-SCLC; (3) No prior systematic treatment for metastatic diseases; however, adjuvant chemotherapy is permissible, provided it was completed at least six months before registration; (4) Performance status (PS) according to the Eastern Cooperative Oncology Group (ECOG) is 0 or 1; (5) Presence of at least one measurable lesion established in accordance with the Response Evaluation Criteria in Solid Tumors (RECIST, version 1.1); (6) Patients not indicated for corticosteroid treatment for central nervous system metastases; (7) No radiotherapy received within seven days before registration; (8) Absence of radiological progression from the end of radiotherapy to the time of registration.

Exclusion criteria: (1) Prior anlotinib therapy received; (2) Previous antineoplastic therapy, including signal transduction inhibitors 28, such as systemic chemotherapy, hormonal, targeted therapy, and endocrine therapy; (3) Diagnosis of other malignant tumors within five years prior to enrollment; (4) Presence of any unresolved toxicity related to treatment at level 2 or higher according to the Common Terminology Criteria for Adverse Events (CTCAE, version 4.0), except for hair loss; (5) Difficulties in oral administration or absorption, including conditions like chronic diarrhea, intestinal obstruction, various dysphagia, and gastrointestinal resection; (6) Asymptomatic central nervous system metastasis; (7) Radiological findings indicating tumor invasion into perivascular tissue or, according to researchers' judgment in subsequent studies, a high likelihood of fatal bleeding due to invasion of vital blood vessels; (8) Uncontrollable pleural, pericardial, or peritoneal effusion requiring repeated drainage.

### Treatments

All patients underwent a treatment regimen involving two consecutive weeks of anlotinib administration (12 mg/day) followed by a one-week break (one treatment session comprised two weeks of treatment and one week of rest). Etoposide (100 mg/m2) was administered intravenously from the 1st to the 3rd day as part of each treatment course. At the beginning of each course, patients received intravenous carboplatin or cisplatin (75 mg/m2), with the calculated area under the concentration-time curve for carboplatin set at 5 mg/ml/min. The treatment protocol included the assessment of tumor response, categorized as stable disease (SD), complete remission (CR), or partial remission (PR). Anlotinib was continued until disease progression, the occurrence of intolerable adverse reactions, or the patient's withdrawal from the study. A total of four chemotherapy courses were administered, involving a combination of etoposide with cisplatin/carboplatin.

### Assessments

We evaluated the therapeutic outcomes using several criteria, including OS, PFS, DCR, and ORR. PFS, often referred to as Progressive Disease (PD)-free survival, measures the time from the experiment's initiation until death from any cause or the onset of PD. In the final follow-up, we examined the number of patients who survived throughout the analysis period. OS, the duration from the registration date until mortality attributed to a specific cause, was assessed, commonly known as the survival duration. Patients who were still alive by the deadline were examined. ORR is determined as the percentage of patients with CR and PR. The establishment of DCR relies on the percentage of patients with CR, PR, and Stable Disease SD. The RECIST (Version 1.1), which evaluates tumor response every two treatment courses, was employed in this study. Patients without brain metastasis and symptoms underwent chest CT, abdominal ultrasonography, and CT/MR every two courses, with brain MR/CT performed at 6-month intervals. For patients with brain metastases, abdominal ultrasound, MR/CT, chest CT, and CT/MR were conducted every two courses. Additionally, patients underwent Positron Emission Tomography (PET) CT and routine brain MR/CT examinations.

### Statistical analysis

The projected increase in the median PFS value is anticipated to be from 4.0 months to 6.5 months. The enrollment period spanned 24 months, with a follow-up duration of 6.5 months. However, owing to the COVID-19 epidemic, progress might have been expedited. Enrollment was concluded due to the IMPAWER133 and Caspian studies, which endorsed the use of chemotherapy plus immunotherapy to enhance survival—typically considered the standard of care.

The summarized data includes the frequency and percentage of categorical variables, as well as the median and range for continuous variables. The calculation of Hazard Ratios (HR) for OS and estimation of PFS were performed using the Kaplan-Meier algorithm. All result measurements are accompanied by 95% confidence intervals (CIs), which are approximated using the Cox proportional hazard model. Differences in patients' baseline clinicopathological characteristics were assessed using either Fisher's exact test or Pearson χ2.

The Log-Rank test was employed in exploratory univariate analysis. To determine statistical significance, the pre-established threshold for testing was set at P < 0.05, indicating a noteworthy distinction. The p-values and CIs for all analyses are two-tailed. Summary statistics for AEs were presented using percentages and frequency counts. IBM SPSS Statistics software (version 24) was utilized for the analysis of all statistical results.

## Results

### Baseline characteristics of clinical subjects

The study was conducted at Linfen Central Hospital from February 2019 to February 2021. A total of 45 previously untreated patients with ES-SCLC were included, and the baseline characteristics of patients in the intention-to-treat set (ITT set; n=45) are presented in **Table 1**. The mean age was 61 years (range: 44-75), with 66.6% of patients being under the age of 65 (30/45). Of the participants, 77.7% were male (35/45), and 71.2% were either past or present smokers (32/45). All 45 patients in the ECOG had a baseline ECOG Performance Status (PS) of 1. The most commonly observed metastatic sites at baseline were the liver (28.9%, 13/45), brain (26.7%, 12/45), bone (28.9%, 13/45), pleura (24.4%, 11/45), and bilateral lungs (24.4%, 11/45). Among them, 20 cases received cisplatin (44.4%, 20/45), and 25 cases received carboplatin (55.5%, 25/45).

Five participants were excluded from the efficacy analysis as they did not complete two courses of treatment, which was necessary for data collection. Despite being under continuous care at a local hospital, these five patients did not undergo efficacy evaluation due to reasons unrelated to the study. The baseline characteristics of patients in the per-protocol set (PP set; n=40) are presented in Table 1. The data indicate that the mean age of the 40 patients was 61 years (range: 44-74), with 62.5% of them being under 65 years old (25/40). Among the participants, 77.5% were male (31/40), and 70.0% were former or current smokers (28/40). The Eastern Cooperative Oncology Group Performance Status (ECOG PS) for all 40 patients was 1 at baseline. The most frequently observed metastatic sites at baseline were the liver (32.5%, 13/40), bone (32.5%, 13/40), brain (30.0%, 12/40), and bilateral lungs (27.5%, 11/40). Of the patients, 42.5% received cisplatin (17/40), and 57.5% received carboplatin (23/40).

### Tumor responses

Table 2 presents the tumor response results. In the PP group, thirty-six patients achieved a PR, constituting approximately 90% of the total SD was observed in 3 cases (7.5%), PD in 2 cases (5.0%). The ORR was 91.0% (95% CI: 78.5%-100.6%), and the DCR was 97.6% (95% CI: 91.8%-103.3%). In the ITT group (n=45), thirty-two patients achieved PR, accounting for approximately 71.1% of the total. Two patients (4.4%) exhibited SD, and one patient (2.2%) reported PD as a response. Nine cases (20.0%) were deemed ineligible for curative effect analysis. The ORR in this group was 75.0% (95% CI: 57.6%-86.4%), and the DCR was 77.8% (95% CI: 64.1%-92.0%).

### Analysis of PFS time for patients in the PP group (n = 40)

**Figure 1:**
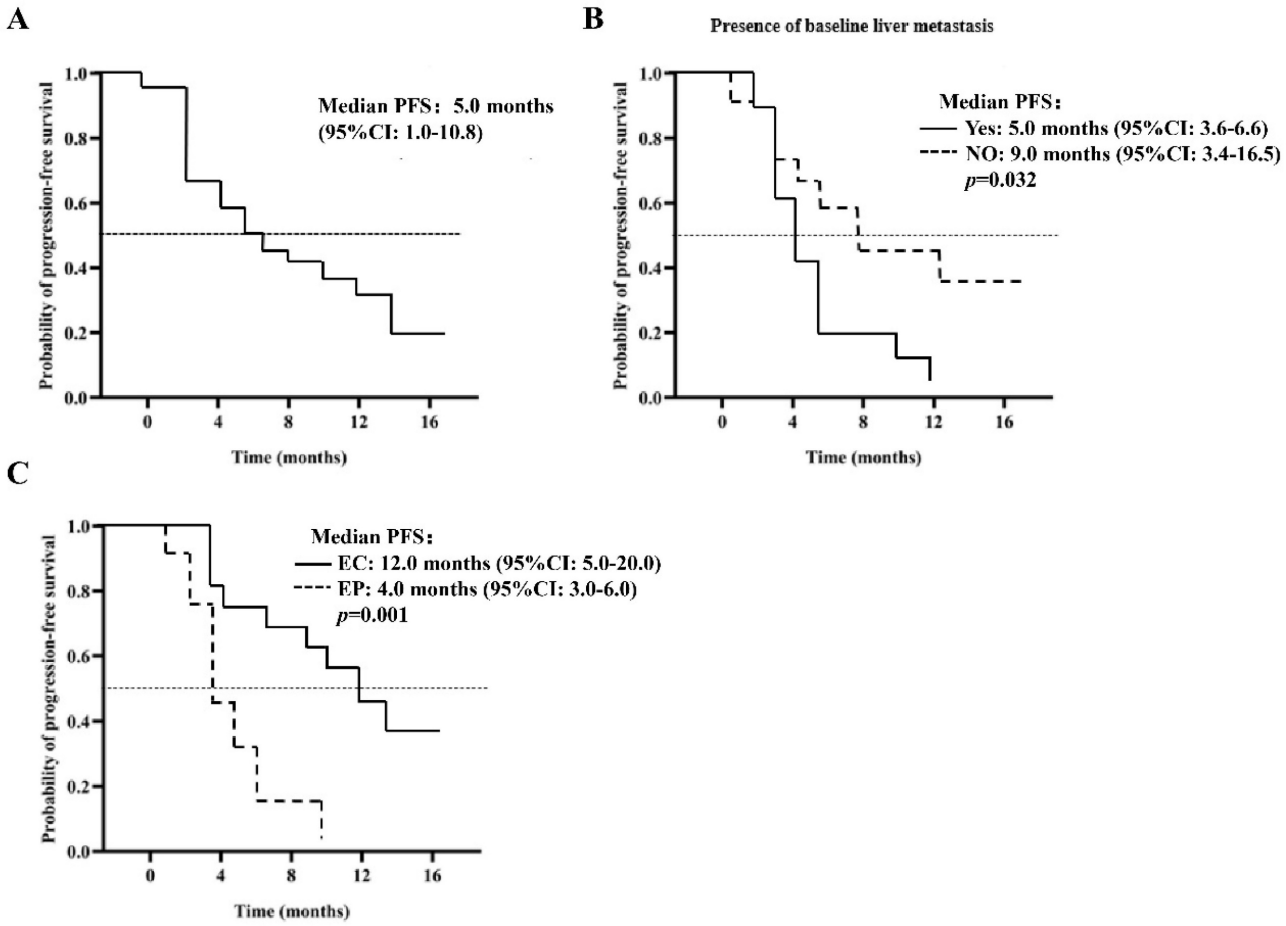
Kaplan Meier curves for PFS (A) 40 patients in the PP group (n = 40); (B) does the patient develop baseline liver metastases; (C) the patient was given EP / EC plus anlotinib.

As of the March 21, 2022 deadline, progression was observed in 27 patients (67.5%). The median PFS is estimated at 5.0 months (95% CI: 1.0-10.8), as illustrated in Figure 1A. Various factors were analyzed to predict PFS, detailed in Table 3. The Log-Rank test revealed a significant association between baseline liver metastasis (p=0.032), chemotherapy regimen (p=0.001), and PFS. The PFS for patients with baseline liver metastasis was notably shorter than that for those with extraneous liver metastasis at baseline (5.0 months, 95% CI: 3.6-6.6 vs. 9.0 months, 95% CI: 3.4-16.5, p=0.032), as depicted in Figure 1B. In multivariate analysis, the chemotherapy regimen emerged as an independent risk factor for PFS (12.0 months, 95% CI: 5.0-20.0 vs. 4.0 months, 95% CI: 3.0-6.0, p=0.021), as shown in Figure 1C. Analysis of 27 patients with PD revealed that 14 (51.8%) received second-line treatment, 10 (37.0%) received the best supportive therapy, and 3 (11.2%) were lost to follow-up.

### Analysis of OS time of patients (PP set)

**Figure 2:**
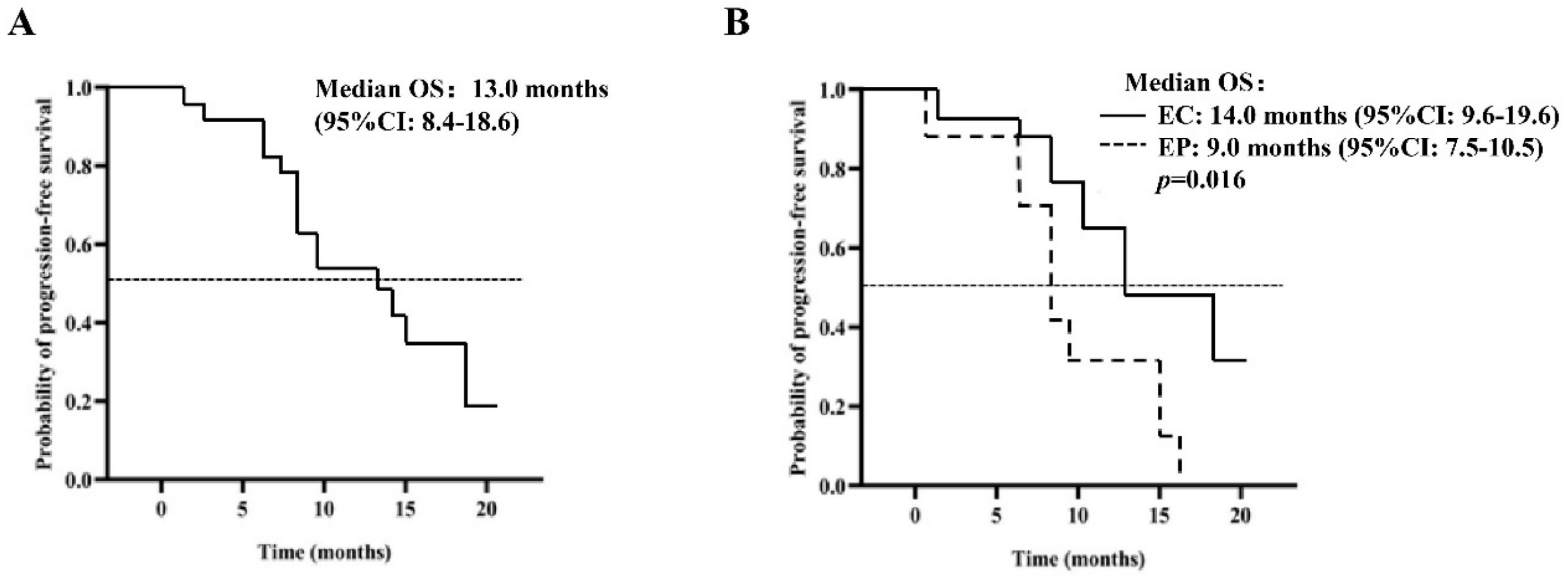
Kaplan-Meier curve of OS (A) 40 patients in the PP group; (B) patients were given EP / EC in combination with anlotinib.

The median OS is estimated to be 13.0 months (95% CI: 8.4-18.6), as depicted in Figure 2A. The analysis, based on the Log-Rank test, indicated a significant correlation between OS and the chemotherapy treatment (p=0.016) (Table 4). Patients who received EP chemotherapy had a substantially shorter OS than those who received EC chemotherapy (EP vs. EC, 9.0 months, 95% CI: 7.5-10.5 vs. 14.0 months, 95% CI: 9.6-19.6, p=0.016) (Figure 2B).

### Report statistics of adverse events and safety in ITT set (n = 45)

The most prevalent AEs were hypertension (24/45, 53.33%), anemia (19/45, 42.22%), alopecia (18/45, 40.00%), elevation of transaminases (11/45, 24.40%), and elevation of alkaline phosphatase (11/45, 24.44%) (Table 5). There were 16 cases of grade 5 adverse reactions (35.56%). No unexpected adverse reactions were observed, and no patient was found to have died as a result of treatment in this study.

## Discussion

This study has significantly contributed to understanding the role of anlotinib combined with etoposide and platinum-based chemotherapy in treating ES-SCLC. Our findings, indicating a median PFS of 5.0 months and OS of 13.0 months, underscore the potential of this combination therapy. These survival rates, coupled with a high DCR and ORR, are promising, especially considering the aggressive nature and traditionally poor prognosis associated with ES-SCLC 29, 30. The primary adverse event observed, hypertension, is a known effect of antiangiogenic therapies and aligns with findings from other studies in lung cancer treatments 31, 32. The manageability of this side effect is crucial for patient care, suggesting that anlotinib can be incorporated into ES-SCLC treatment protocols without significantly increasing treatment-related toxicity 33.

The rapid progression and development of chemotherapy resistance in ES-SCLC remain substantial challenges in oncology 34. Our study takes a novel approach by introducing anlotinib in first-line treatment, providing a potential avenue to enhance outcomes in ES-SCLC patients. This strategy aligns with the evolving landscape of lung cancer treatment, where integrating novel agents with traditional therapies is increasingly recognized as a viable approach to overcoming treatment resistance and improving survival rates 35, 36. While NSCLC has witnessed substantial advancements with targeted therapies and immunotherapies, ES-SCLC has not experienced the same level of therapeutic evolution due to its unique biological characteristics 37, 38. Our study, focusing on anlotinib for ES-SCLC, contributes to bridging this gap, suggesting that targeted approaches can yield positive outcomes in this challenging cancer subtype 39.

Our findings suggest that anlotinib's antiangiogenic properties may offer a distinct advantage in treating ES-SCLC. Angiogenesis plays a crucial role in tumor growth and metastasis, and its inhibition could be particularly effective in combating the rapid progression characteristic of ES-SCLC 40. This hypothesis aligns with the observed efficacy in our study, where anlotinib, combined with chemotherapy, led to significant tumor control and extended survival times.

Further, the observed DCR and ORR in our study are notable. The high response rates suggest that anlotinib, combined with etoposide and platinum-based chemotherapy, can induce substantial tumor regression, an essential factor in managing ES-SCLC 41. This is particularly relevant given the often-limited response of ES-SCLC to conventional chemotherapy after initial treatment 42.

The potential for anlotinib in ES-SCLC raises questions about the optimal integration of targeted therapies in the standard treatment regimen for this cancer subtype. Our results suggest that earlier introduction of targeted agents like anlotinib in the treatment protocol could be beneficial. This approach could potentially transform the current therapeutic strategy for ES-SCLC, where advancements have been slower than other lung cancer subtypes 43, 44.

Furthermore, the implications of our findings extend beyond the immediate scope of ES-SCLC treatment. They highlight the importance of continuing to explore and understand the molecular pathways involved in ES-SCLC. Investigating the mechanisms through which anlotinib exerts its anti-cancer effects could lead to discovering novel biomarkers for predicting treatment response and identifying patients who would most benefit from this therapy 45.

In conclusion, our study presents anlotinib combined with etoposide and platinum-based chemotherapy as a promising new treatment option for newly diagnosed ES-SCLC—our study's favorable survival outcomes and manageable safety profile warrant further investigation. Larger-scale, randomized controlled trials are essential to confirm these findings. Such studies will be crucial in establishing a new standard of care for ES-SCLC, offering hope for improved treatment outcomes in this challenging and aggressive form of lung cancer 46.

## Conclusion

In general, our results demonstrate the efficacy and assurance of anlotinib combined with chemotherapy for managing newly diagnosed ES-SCLC. In the future, numerous randomized phase III clinical trials will be necessary to further validate the therapeutic effectiveness of anlotinib in the treatment of ES-SCLC.

## Figures and Tables

**Table 1:** Baseline characteristics of patients with ITT (n= 45) set and PP set (n= 40)

Characteristics	ITT set	PP set	P value
Age, years			
Mean	61 (44-75)	61 (44-74)	0.76
Age group			
<65 years	30 (66.6%)	25 (62.5%)	0.43
≥65 years	15 (33.4%)	15 (37.5%)	0.85
Smoking history			
Never-smoker	13 (28.8%)	12 (30.0%)	0.33
Former or current smoker	32 (71.2%)	28 (70.0%)	0.35
Sex			
Male	35 (77.7%)	31 (77.5%)	0.91
Female	10 (22.3%)	9 (22.5%)	0.57
ECOG PS at baseline			
1	45 (100.0%)	40 (100.0%)	0.68
Metastatic sites at baseline			
Bilateral lung	11 (24.4%)	11 (27.5%)	0.66
Liver	13 (28.9%)	13 (32.5%)	0.74
Bone	13 (28.9%)	13 (32.5%)	0.15
Brain	12 (26.7%)	12 (30.0%)	0.26
Supraclavicular lymph node	9 (20.0%)	8 (20.0%)	0.39
Adrenal gland	6 (13.3%)	6 (15.0%)	0.66
Pleural	11 (24.4%)	8 (20.0%)	0.19
Others	13 (28.9%)	8 (20.0%)	0.78
Chemotherapy			
EP	20 (44.4%)	17 (42.5%)	0.49
EC	25 (55.5%)	23 (57.5%)	0.83

**Table 2:** Therapeutic efficacy and tumor response of ITT set (n= 45) and PP set (n= 40)

Responses	ITT set (n= 45) [n or %]	PP set (n= 40) [n or %]	P values
PR	32 (71.1)	36 (90.0)	0.84
PD	1 (2.2)	2 (5.0)	0.22
Ineligible	9 (20.0)	0 (0.0)	0.15
SD	2 (4.4)	3 (7.5)	0.32
DCR	77.8% [95% CI: 64.1% - 92.0%]	97.6% [95% CI: 91.8% - 103.3%]	0.58
ORR	75.0% [95% CI: 57.6% - 86.4%]	91.0% [95% CI: 78.5% - 100.6%]	0.92

**Table 3:** Evaluation of PFS of PP set by Cox multivariate analysis

		Multivariate analysis		
Variables	Log-rank test	HR	95% CI	p-value
Age (<65 vs. ≥ 65 years)	0.061	0.432	0.412-1.264	0.254
Lung metastasis (yes vs. no)	0.373	0.961	0.865-2.654	0.425
Adrenal gland metastasis (yes vs. no)	0.672	0.857	0.236-0.935	0.234
Bone metastasis (yes vs. no)	0.187	1.067	0.968-3.935	0.135
Pleural metastasis (yes vs. no)	0.259	1.265	0.868-1.896	0.785
Smoking history (yes vs. no)	0.314	0.587	0.457-0.824	0.657
Liver metastasis (yes vs. no)	**0.032**	0.587	0.312-1.884	0.531
Sex (male vs. female)	0.897	0.256	0.157-0.482	0.681
Brain metastasis (yes vs. no)	0.471	0.759	0.582-0.897	0.279
Chemotherapy (EP vs. EC)	**0.001**	4.367	1.457-11.89	**0.021**
Supraclavicular lymph node metastasis (yes vs. no)	0.453	2.215	1.256-4.658	0.658

**Table 4:** Evaluation of OS of PP set by Cox multivariate analysis

Variables	Log-rank test
Brain metastasis (yes vs. no)	0.772
Sex (male vs female)	0.714
Age (<65 vs. ≥65 years)	0.223
Bone metastasis (yes vs. no)	0.451
Lung metastasis (yes vs. no)	0.670
Smoking history (yes vs. no)	0.891
Supraclavicular lymph node metastasis (yes vs. no)	0.278
Chemotherapy (EP vs. EC)	**0.016**
Adrenal gland metastasis (yes vs. no)	0.217
Pleural metastasis (yes vs. no)	0.289
Liver metastasis (yes vs. no)	0.254

**Table 5:** Report statistics of AEs in ITT set (n= 45)

AEs	All grades [no. (%)]	≥Grade 3 [no. (%)]
Any	43(95.56%)	16(35.56%)
Anemia	19(42.22%)	
Hypertension	24(53.33%)	2(4.44%)
Alopecia	18(40.00%)	
Elevated alkaline phosphatase	11(24.44%)	
Neutropenia	7(15.56%)	6(13.33%)
Leukopenia/hyponatremia	9(20.00%)	
Elevated transaminases	11(24.40%)	
Thrombocytopenia	10(22.22%)	
Fatigue	6(13.33%)	5(11.11%)
Elevated white blood cells	5(11.11%)	
Oral pain	5(11.11%)	
Diarrhea	4(8.89%)	
Constipation	4(8.89%)	
Nausea	4(8.89%)	
Rash	4(8.89%)	1(2.22)
Thrombosis	2(4.44%)	
Hyperuricemia	2(4.44%)	
Hemorrhage	2(4.44%)	
Vomiting	1(2.22%)	
Hyperkalemia	1(2.22%)	
Hypocalcemia	1(2.22%)	1(2.22)
Decreased appetite	1(2.22%)	
Hypophosphatemia	1(2.22%)	1(2.22)
